# Supplementary material for: Natural killer cells modulate motor neuron-immune cell cross talk in models of Amyotrophic Lateral Sclerosis
Source: Nat Commun. 2020 Apr 14;11:1773. doi: 10.1038/s41467-020-15644-8 (PMC7156729; doi:10.1038/s41467-020-15644-8)
Supplement: Supplementary file 1 — Supplementary Information [file 41467_2020_15644_MOESM1_ESM.pdf]

Supplementary information for the manuscript:

Natural killer cells modulate motor neuron-immune cell cross talk in models of Amyotrophic Lateral Sclerosis

Garofalo et al.

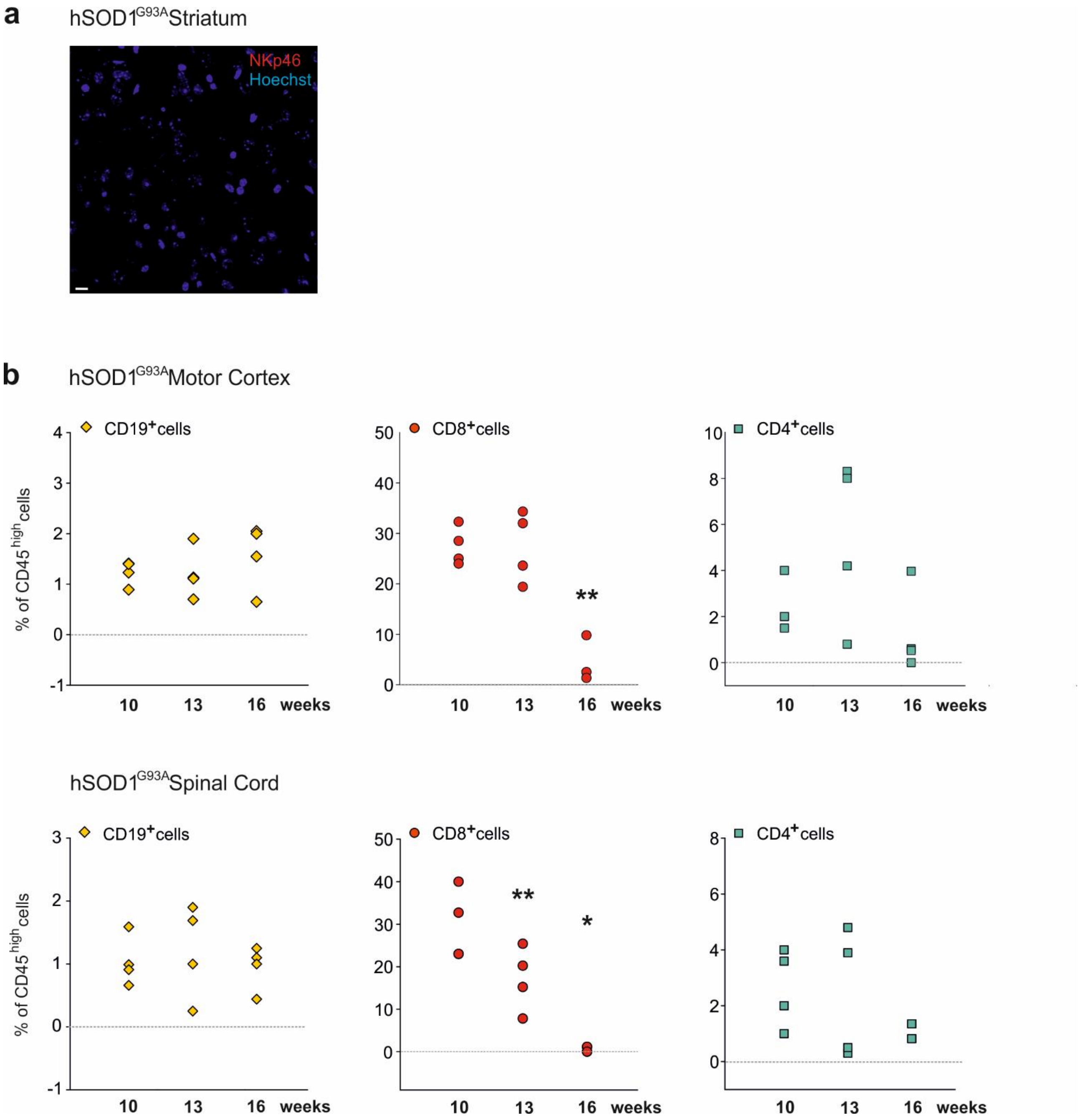

Supplementary Figure 1: Analysis of immune cells infiltrated in CNS of hSOD1^{G93A} mice. (**a**) Representative image of the striatum of hSOD1^{G93A} mice, stained for NKp46 (n=5). Scale bar: 20 µm. (**b**) Time course analysis of CD19^{+}/ CD45^{+}, CD8^{+}/CD3^{+}/CD45^{+}, and CD4^{+}/CD3^{+}/CD45^{+} cells frequency in the cerebral motor cortex and lumbar spinal cord of hSOD1^{G93A} mice (dotted lines indicate values in wt mice, n = 4; * P <0.05, ** P < 0.01 power 0.997 *vs* 10 week-old hSOD1^{G93A} mice, one-way ANOVA; values of the three cell populations in hSOD1^{G93A} mice were all significantly different from wt mice and were not indicated in the figures, P < 0.01, power 1, one-way ANOVA).

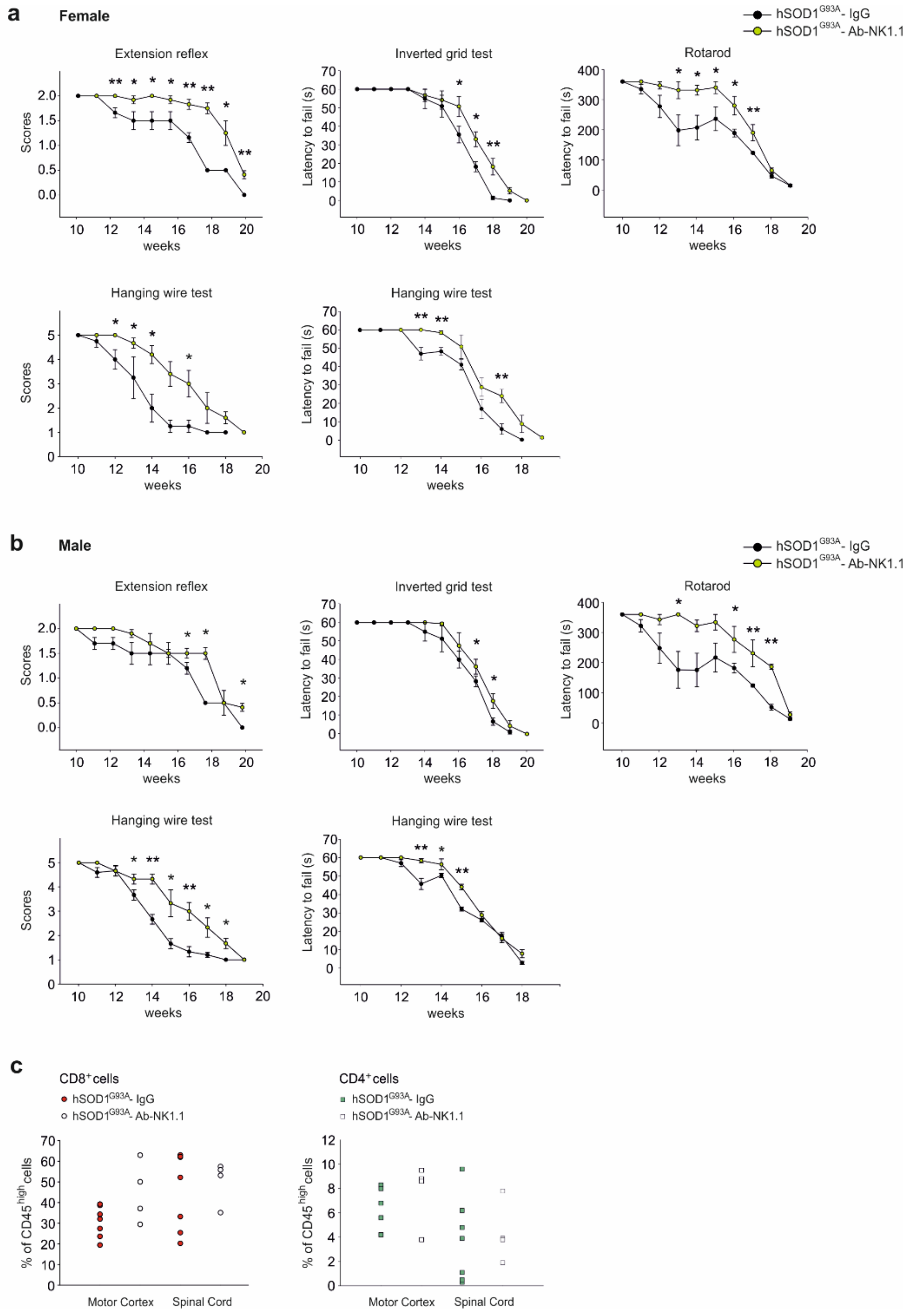

Supplementary Figure 2: NK cell depletion improves behavioural motor symptoms in hSOD1^{G93A} mice. Analyses of motor function in female (**a**) and male (**b**) hSOD1^{G93A} mice treated with Ab-NK1.1 or vehicle as: extension reflex, inverted grid test, rotarod test and hanging wire test. Behavioural tests were performed once a week, starting from 8 weeks (female: n = 7 and male: n= 4 mice per treatment. Data are expressed as mean ± s.e.m. * P <0.05, ** P < 0.01, power >0.9 *vs* vehicle mice, two-tailed Student's t-test). (**c**) Analysis of CD8^{+}/CD3^{+}/CD45^{+} and CD4^{+}/CD3^{+}/CD45^{+} cells frequency in the cerebral motor cortex and lumbar spinal cord of 13 week-old hSOD1^{G93A} mice (wt: n = 6 and hSOD1^{G93A} : n=4).

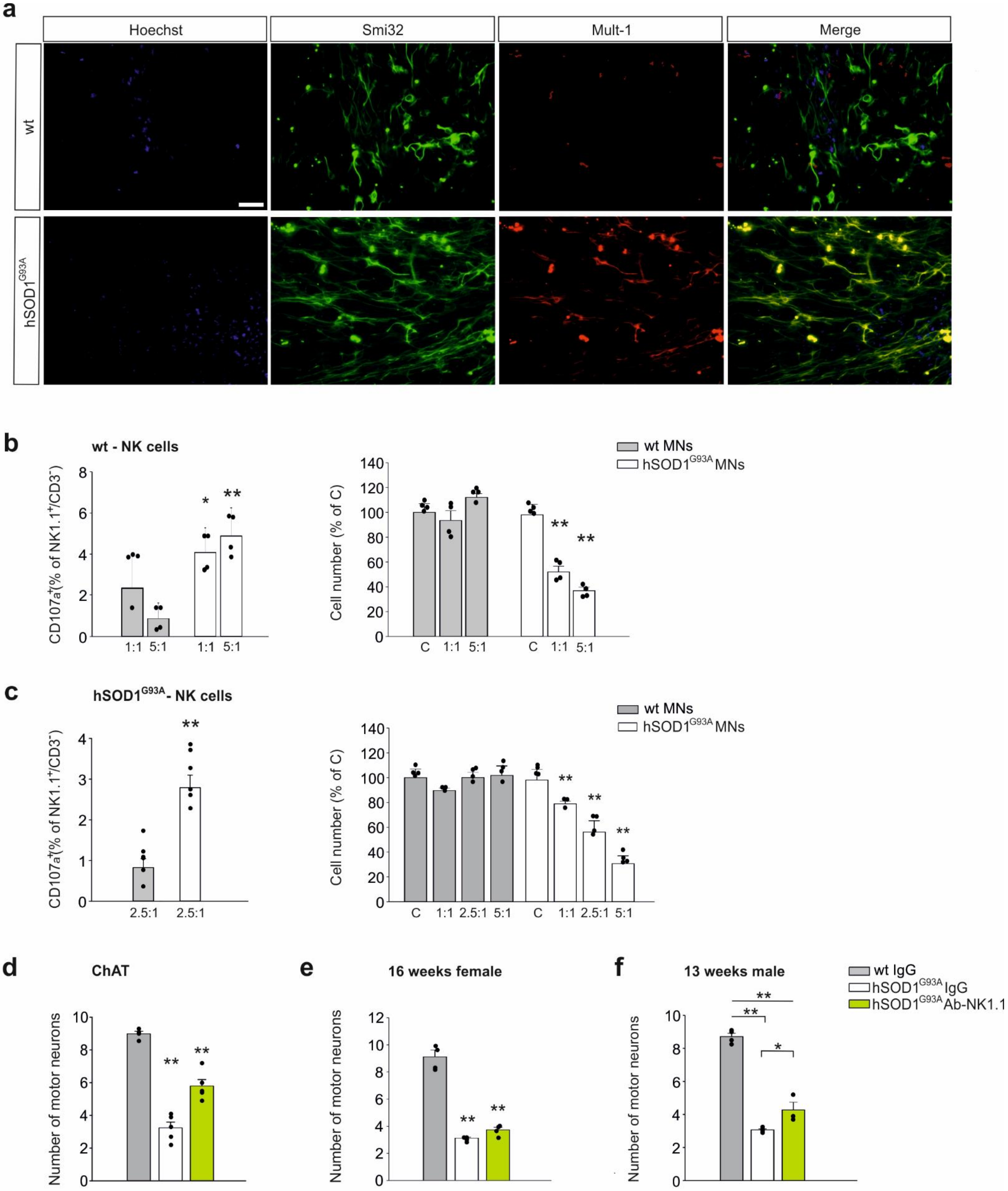
a
Hoechst
Smi32
Mult-1
Merge
wt
hSOD1G93A
b
wt - NK cells
CD107a+(% of NK1.1+/CD3-)
1:1 5:1 1:1 5:1
wt MNs
hSOD1G93A MNs
Cell number (% of C)
C 1:1 5:1 C 1:1 5:1
c
hSOD1G93A- NK cells
2.5:1 2.5:1
C 1:1 2.5:1 5:1 C 1:1 2.5:1 5:1
d
ChAT
Number of motor neurons
e
16 weeks female
f
13 weeks male
wt IgG
hSOD1G93A IgG
hSOD1G93A Ab-NK1.1

Supplementary Figure 3: NK cell – MN communication in ALS. (**a**) Expression of Mult-1 in primary MN cultures obtained from wt or hSOD1^{G93A} (13 weeks) mice (n = 4). Scale bar: 50 μm. (**b**) NK cells, isolated from the spleen of wt mice, were incubated at different E:T ratios (5:1, 1:1) with primary neuronal cultures isolated from the spinal cord of wt or hSOD1^{G93A} (13 weeks) mice, and degranulation of NK cells (having subtracted basal degranulation) was assessed by FACS analysis of CD107a^{+} cells (left panel) (n = 4, ** $P < 0.01$, power 0.997, one-way ANOVA). Cell viability of the same neuronal cultures is shown in the right panel (n = 4, ** $P < 0.01$, power 0.971, one-way ANOVA). (**c**) Cell viability in neuronal cultures obtained from the spinal cord of wt or hSOD1^{G93A} mice (13weeks) upon incubation with NK cells isolated from the spleen of hSOD1^{G93A} mice at different E:T ratios (5:1, 2.5:1, 1:1). Degranulation of hSOD1^{G93A} -NK cells was assessed by FACS analysis of CD107a^{+} cells (left panel) (wt: n = 5 and hSOD1^{G93A} : n= 6 for CD107a analysis, wt: n = 4 and hSOD1^{G93A} : n= 4 cell numbers, ** $P < 0.01$ power 995 one-way ANOVA). (**d**) Quantification of MNs (counted as ChAT^{+} cells in the ventral horns of the spinal cord) in wt and hSOD1^{G93A} mice (16 weeks), treated with vehicle or Ab-NK1.1. (n = 5. Data are shown as mean ± s.e.m. ** $P < 0.01$ power 1, one-way ANOVA). (**e**) Quantification of MNs (counted as Smi32^{+} cells in the ventral horns of the spinal cord) in wt and hSOD1^{G93A} mice (16weeks), treated with vehicle or Ab-NK1.1. (n = 4. Data are shown as mean ± s.e.m. ** $P < 0.01$ power 1, one-way ANOVA). (**f**) Quantification of MNs (counted as Smi32^{+} cells in the ventral horns of the spinal cord) in wt and hSOD1^{G93A} male mice (13weeks) treated with vehicle or Ab-NK1.1 (wt: n=3;, hSOD1^{G93A} vehicle: n=5; hSOD1^{G93A} Ab-NK1.1: n=3. Data are shown as mean ± s.e.m. ** $P < 0.01$ power 1, one-way ANOVA). Error bars show mean ± SEM.

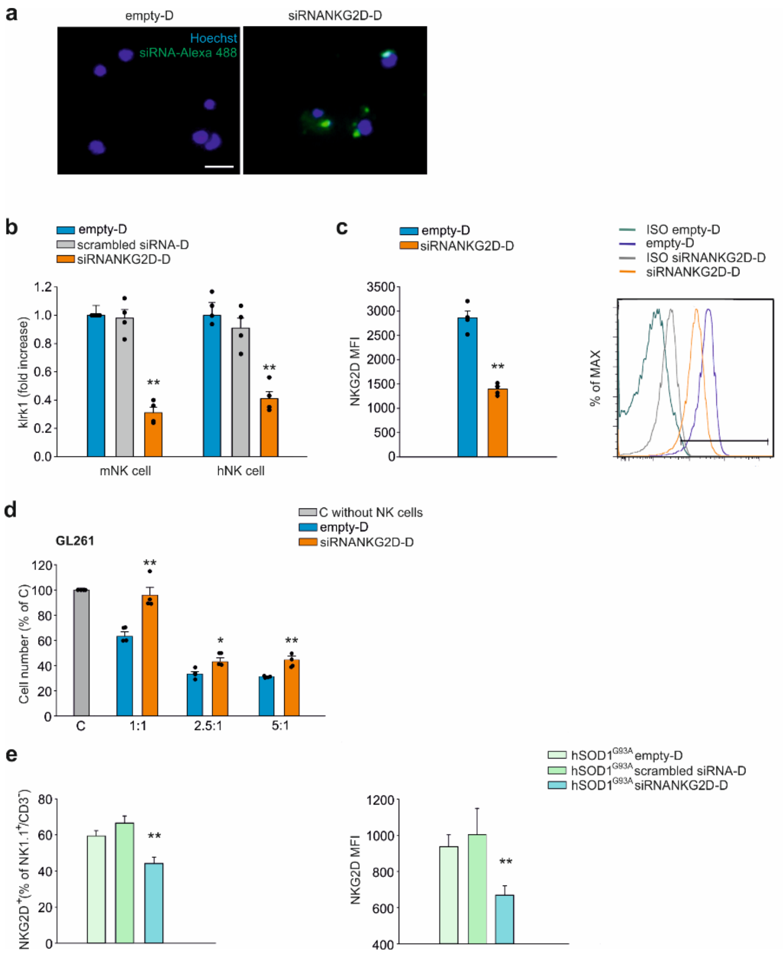

Supplementary Figure 4: Dendrimers-mediated siRNA delivery in murine and human primary NK cells. (**a**) Immunofluorescence detection of dendrimers or dendrimers-siRNA-488 Alexa fluor uptake in murine NK cells after 48 h of incubation (n=3). Scale bar: 5 µm. (**b**) RT-PCR of *klrk1* gene in human and murine NK cells treated with 200nM siRNA (n = 4, data are expressed as mean ± s.e.m. ** P < 0.01 vs empty dendrimers, one-way ANOVA). (**c**) Detection of NKG2d protein in murine NK cells transfected with 200nM of siRNA-NKG2d. Protein expression was assessed by FACS 48h after transfection. (n = 4, data are expressed as mean ± s.e.m. ** P < 0.01 vs empty dendrimers, one-way ANOVA). (**d**) Murine NK cells were incubated at different E:T ratios (5:1, 2.5:1, 1:1) with GL261 murine glioma cell line. GL261 viability was assessed after 48 h of incubation with NK cells (n = 4, ** P < 0.01 power 1, one-way ANOVA). (**e**) The expression and the MFI of NKG2D was assessed in NK cells isolated from the peripheral blood of hSOD1^{G93A} mice (13 weeks) treated with dendrimers or dendrimers-loaded with siRNA-NKG2D (n=4 mice per treatment, data are expressed as mean ± s.e.m. ** P < 0.01 one-way ANOVA).

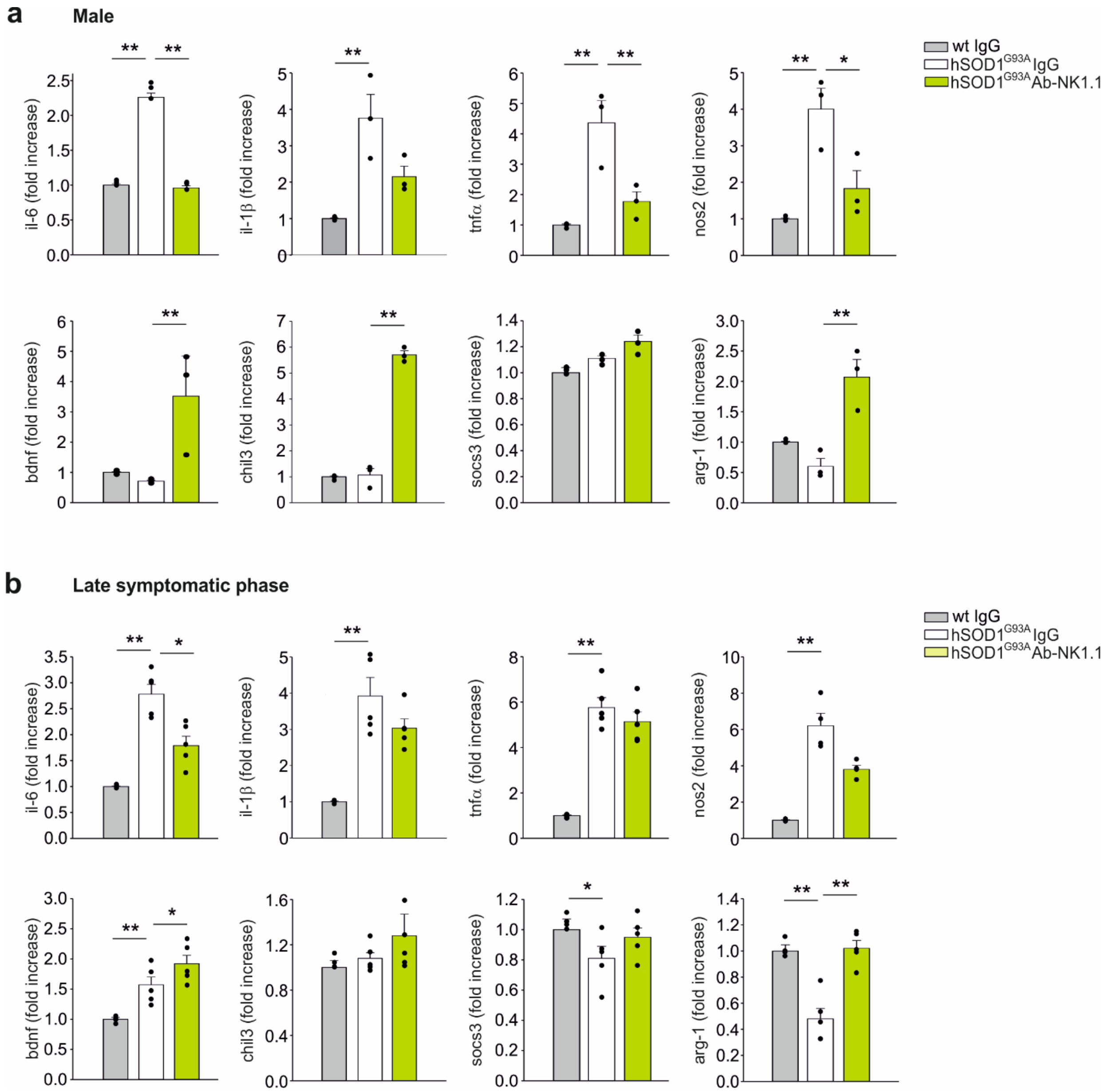

Supplementary Figure 5: Gene expression analysis in hSOD1^{G93A} mice. (**a**) RT-PCR of *il-6, il-1β, tnfα, nos2*, and *bdnf, chil3, socs3, arg-1* gene expression in microglial cells isolated from the lumbar spinal cord of wt (n= 4) or hSOD1^{G93A} (n=4) male mice (13 weeks) treated with vehicle or Ab-NK1.1. Data are the mean ± s.e.m. wt vs hSOD1^{G93A} and hSOD1^{G93A} vs hSOD1^{G93A}Ab-NK1.1 * P < 0.05 ** P < 0.01 power >9, one-way ANOVA). (**b**) RT-PCR of *il-6, il-1β, tnfα, nos2*, and *bdnf, chil3, socs3, arg-1* gene expression in microglia isolated from the lumbar spinal cord of wt or hSOD1^{G93A} mice (16weeks) treated with vehicle or Ab-NK1.1 (n = 5 mice. Data are the mean ± s.e.m. wt vs hSOD1^{G93A} and hSOD1^{G93A} vs hSOD1^{G93A}Ab-NK1.1; * P < 0.05 ** P < 0.01 power>9, one-way ANOVA).

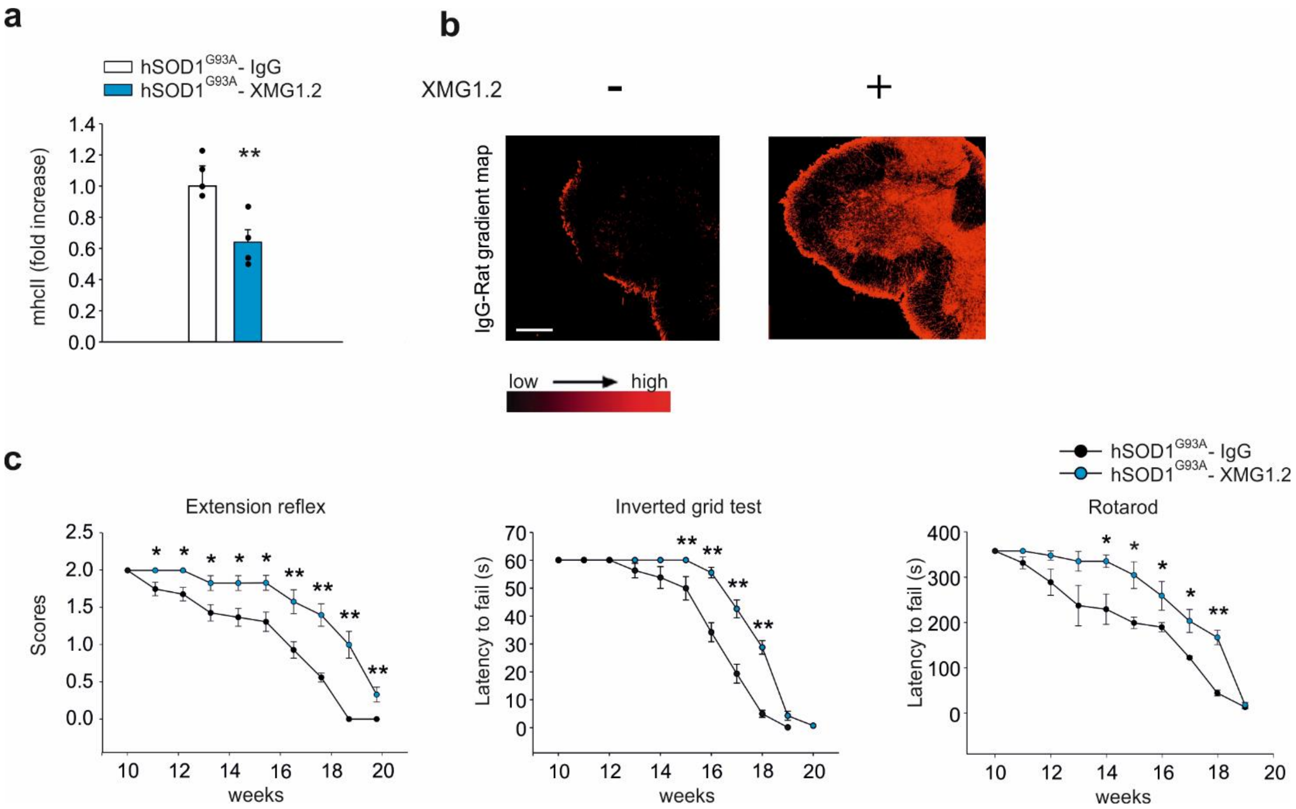

Supplementary Figure 6: Effect of IFNγ inhibition in motor behaviour in hSOD1^{G93A} mice. (**a**) RT-PCR of *mhcII* expression in microglia isolated from hSOD1^{G93A} mice treated with vehicle or XMG1.2. (n = 4, ** P < 0.01 power 0.971, two-tailed Student's *t*-test). Error bars show mean ± SEM. (**b**) Representative immunofluorescence images of IgG-Rat in the spinal cord of vehicle (left) or XMG1.2 treated (right) hSOD1^{G93A} mice (n=4, Scale bar: 200 μm). (**c**) Motor function analysis in hSOD1^{G93A} mice treated with XMG1.2 (n=6) or vehicle (n=7) as: extension reflex (left), inverted grid test (center), rotarod test (right). Behavioural tests were performed once a week, starting from 8weeks (Data are expressed as mean $\pm$ s.e.m. * P <0.05, ** P < 0.01, power >9 *vs* vehicle mice, two-tailed Student's *t*-test).

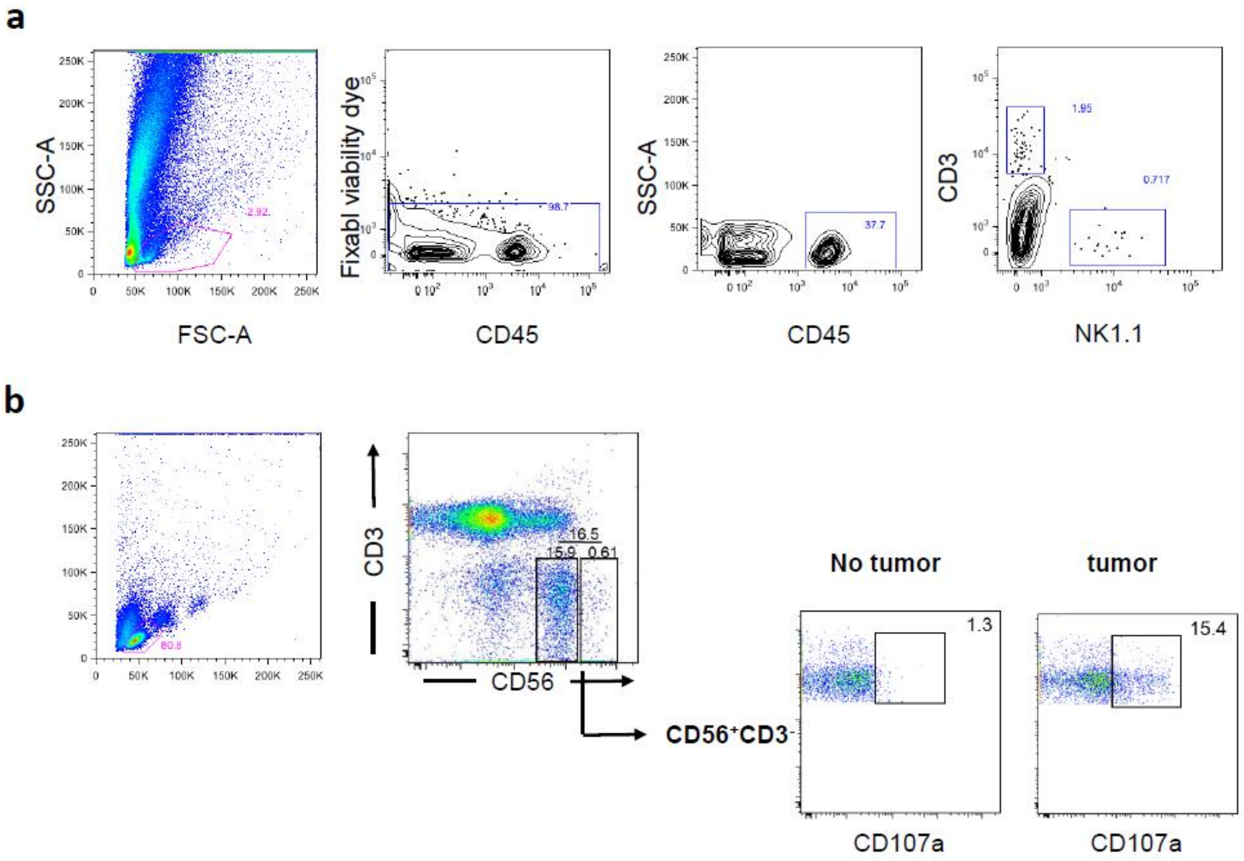

Supplementary Figure 7: Gating strategies. (**a**) Hematopoietic cells were purified from mouse spinal cord by percoll (40%) and were labeled with fixable/viability dye to exclude death cells and with anti-CD45, -CD3 and -NK1.1. NK cells were identified as $CD3^-$/$NK1.1^+$ cells among $CD45^+$ cells. (**b**) Mononuclear cells were isolated from blood samples of ALS patients by lympholyte and stained with anti-CD3 and -CD56 mAbs. Lymphocytes were morphologically gated and total NK cells were identified as $CD3^-$/$CD56^+$ cells and further divided into $CD56^{bright}$ and $CD56^{dim}$ cells. An example of analysis of NK cell degranulation is shown below: after incubation with or without tumor cells, NK cells were stained with anti-CD3 and –CD56 and anti-CD107a mAbs and analysis of $CD107a^+$ cells was performed among total $CD3^-$/$CD56^+$ NK cells.

Genes*	Forward (5’- 3’)	Reverse (5’- 3’)
ulbp1	CTCATAGGAACAGCATGA	TCCTGTGAAATGTTTGTC
rae-1	ACCCGAATGCAGACAGGAAGTTGA	GGACCTTGAGGTTGATCTTGGCT
nectin-2	GTCCTTCGTCTCTGCCAAGCA	CACTGCGTGGATGACCAGCTG
pvr	GAGGTGACGCATGTGTCACAG	TCTTGCCGTCCACCTGGCTTG
il-1β	GCAACTGTTCCTGAACTCAACT	ATCTTTTGGGGTCCGTCAACT
il-6	GATGGATGCTACCAAACTGGA	TCTGAAGGACTCTGGCTTTG
tnfα	GTGGAACTGGCAGAAGAG	CCATAGAACTGATGAGAGG
nos2	ACATCGACCCGTCCACAGTAT	CAGAGGGGTAGGCTTGTCTC
nox2	TGAATGCCAGAGTCGGGATTT	CGAGTCACGGCCACATACA
p47phox	TCCCAACTACGCAGGTGAAC	CCTGGGTTATCTCCTCCCCA
chil3	CAGGTCTGGCAATTCTTCTGAA	GTCTTGCTCATGTGTGTAAGTGA
socs3	GCTCCAAAAGCGAGTACCAGC	AGTAGAATCCGCTCTCCCTGCAG
arg-1	CTCCAAGCCAAAGTCCTTAGAG	AGGAGCTGTCATTAGGGACATC
il-15	CATCCATCTCGTGCTACTTGTGTT	CATCTATCCAGTTGGCCTCTGTTT
bdnf	TGAGTCTCCAGGACAGCAAA	TGTCCGTGGACGTTTACTTCT
tgfβ	GGAGAGCCCTGGATACCAAC	AAGTTGGCATGGTAGCCCTT
msod1	CCAGTGCAGGACCTCATTTT	CACCTTTGCCCAAGTCATCT
hsod1	CACGTGGGCTCCAGCATT	TCACCAGTCATTTCTGCCTTTG
ccl2	AGGTCCCCTGTCATGCTTCTG	TCTCCAGCCTACTCATTGGG
p2yr12	CCTGTCGTCAGAGACTACAAG	GGATTTACTGCGGATCTGAAAG
trem2	ATGGGACCTCTCCACCAGTT	TCACGTACCTCCGGGTCCA
kcnn4	GGCTGAAACACCGGAAGCTC	CAGCTCTGTCAGGGCATCCA
foxp3	CCCAGGAAAGACAGCAACCTT	TTCTCACAACCAGGCCACTTG
klrk1	TACTGTGGCCCATGTCCTAA	CTTTCAGAAGGCTGGCATTT
gapdh	TCGTCCCGTAGACAAAATGG	TTGAGGTCAATGAAGGGGTC

Supplementary Table 1: Primers used for qPCR analysis

*Gene names are in italic.
