# Supplementary material for: Natural killer cells modulate motor neuron-immune cell cross talk in models of Amyotrophic Lateral Sclerosis
Source: Nat Commun. 2020 Apr 14;11:1773. doi: 10.1038/s41467-020-15644-8 (PMC7156729; doi:10.1038/s41467-020-15644-8)
Supplement: Supplementary file 3 — Reporting Summary [file 41467_2020_15644_MOESM3_ESM.pdf]

nature research

Corresponding author(s): Cristina Limatola and Stefano Garofalo

Last updated by author(s): 21/02/2020

Reporting Summary

Nature Research wishes to improve the reproducibility of the work that we publish. This form provides structure for consistency and transparency in reporting. For further information on Nature Research policies, see Authors & Referees and the Editorial Policy Checklist .

Statistics

For all statistical analyses, confirm that the following items are present in the figure legend, table legend, main text, or Methods section.

n/a	Confirmed	
☐	☒	The exact sample size (*n*) for each experimental group/condition, given as a discrete number and unit of measurement
☐	☒	A statement on whether measurements were taken from distinct samples or whether the same sample was measured repeatedly
☐	☒	The statistical test(s) used AND whether they are one- or two-sided<br>*Only common tests should be described solely by name; describe more complex techniques in the Methods section.*
☐	☒	A description of all covariates tested
☐	☒	A description of any assumptions or corrections, such as tests of normality and adjustment for multiple comparisons
☐	☒	A full description of the statistical parameters including central tendency (e.g. means) or other basic estimates (e.g. regression coefficient) AND variation (e.g. standard deviation) or associated estimates of uncertainty (e.g. confidence intervals)
☒	☐	For null hypothesis testing, the test statistic (e.g. *F*, *t*, *r*) with confidence intervals, effect sizes, degrees of freedom and *P* value noted<br>*Give P values as exact values whenever suitable.*
☒	☐	For Bayesian analysis, information on the choice of priors and Markov chain Monte Carlo settings
☒	☐	For hierarchical and complex designs, identification of the appropriate level for tests and full reporting of outcomes
☐	☒	Estimates of effect sizes (e.g. Cohen's *d*, Pearson's *r*), indicating how they were calculated

Our web collection on statistics for biologists contains articles on many of the points above.

Software and code

Policy information about availability of computer code

Data collection	Analyze Skeleton plugin; MetaMorph 7.6.5.0; ImageJ. 1.52t; FlowJo 9.3.2.
Data analysis	Statistical analyses comprising calculation of degrees of freedom were done using Sigma Plot 11.0, Imaris; Origin 7, and Prism 7 software.

For manuscripts utilizing custom algorithms or software that are central to the research but not yet described in published literature, software must be made available to editors/reviewers. We strongly encourage code deposition in a community repository (e.g. GitHub). See the Nature Research guidelines for submitting code & software for further information.

Data

Policy information about availability of data

All manuscripts must include a data availability statement. This statement should provide the following information, where applicable:

- Accession codes, unique identifiers, or web links for publicly available datasets
- A list of figures that have associated raw data
- A description of any restrictions on data availability

The data that support the findings of this study are available from the corresponding author upon reasonable request. The source data refer to Figures. 1b, 4a, 7f,g,i.

Field-specific reporting

Please select the one below that is the best fit for your research. If you are not sure, read the appropriate sections before making your selection.

☒ Life sciences ☐ Behavioural & social sciences ☐ Ecological, evolutionary & environmental sciences

Life sciences study design

All studies must disclose on these points even when the disclosure is negative.

Sample size	The sample size (n) was chosen differently for experiments of animal survival, marker expression, etc.. considering the following relation: n≥2sigma(Zalpha/D)2 where sigma is substituted by an extimate of variance (s2); alpha is at 0.05 (and Zalpha=-2) and D is the difference among treatments. Power analysis is always indicated.
Data exclusions	Animals considered for the analysis were selected for age and symptomatic stage of the disease. We did not exclude animals or data from the study.
Replication	All attemps to replicate the results were successful.
Randomization	For experiments aimed at exploring the role of NK cells or IFNg in ALS progression, animals were randomly chosen among different colonies before the treatments with the differents drugs.
Blinding	The investigators performing motor test, RT-PCR, immunofluorescence, FACS, etc analyses where blinded to group allocation; they always received the samples from a third laboratory member, not directly involved in data analysis.

Reporting for specific materials, systems and methods

We require information from authors about some types of materials, experimental systems and methods used in many studies. Here, indicate whether each material, system or method listed is relevant to your study. If you are not sure if a list item applies to your research, read the appropriate section before selecting a response.

Materials & experimental systems

n/a	Involved in the study	
☐	☒	Antibodies
☐	☒	Eukaryotic cell lines
☒	☐	Palaeontology
☐	☒	Animals and other organisms
☐	☒	Human research participants
☒	☐	Clinical data

Methods

n/a	Involved in the study	
☒	☐	ChIP-seq
☐	☒	Flow cytometry
☒	☐	MRI-based neuroimaging

Antibodies

Antibodies used	Antibodies used for immunofluorescence studies include: goat anti-ChAT (#AB144P) Ab (1:100 Merck Millipore); hamster anti-Mult-1 Ab (Cat# 12-5863-81) (1:100 Life technologies Invitrogen); rabbit anti-Iba1 (1:200 Wako) (Cat# 019-19741); mouse anti-ULBP-3 Ab (Cat# MAB1517) (1:100 R&D systems); rat anti-Foxp3 Ab (Clone: FJK-16s) (Cat# 53-5773-82) (1:100 Thermo Fisher); rat anti-CD31 Ab (#3568S) (1:200 Cell Signaling); mouse anti-SMI-32 Ab (1:500 BioLegend); rabbit anti-NKp46 (M20) #sc-18161 (1:50 Santa Cruz).<br>Antibodies used for in vivo depletion include: anti-CCL2 (0.2 mg clone: 2H5) (Cat# BE0185), anti-NK1.1 (0.2 mg Cat# BE0036), anti XMG1.2 (0.2 mg), all from Bioxcell.
Validation	The antibodies were validated according to the manufacturer.

Eukaryotic cell lines

Policy information about cell lines

Cell line source(s)	GL261 murine glioma cell line was obtained from ATCC.
Authentication	None of the cell lines were authentiicated.
Mycoplasma contamination	Cells were negative for mycoplasma contamination.
Commonly misidentified lines<br>(See ICLAC register)	No cell lines used in this study were found in the database of commonly misidentified cell lines that is maintained by ICLAC register.

Animals and other organisms

Policy information about studies involving animals; ARRIVE guidelines recommended for reporting animal research

Laboratory animals	hSOD1G93A B6.Cg-Tg(SOD1-G93A)1Gur/J line female and male 8-week-old; TDP43A315T [B6.Cg-Tg(Prnp-TARDBP*A315T) 95Balo/J] female 8-week-old; C57BL/6-Prf1<tm1Sdz>/J female 8-week-old; non-transgenic C57BL/6J female and male mice 8-week-old.
Wild animals	The study did not involve wild animals.
Field-collected samples	No field-collected samples were used.
Ethics oversight	Experiments described in the present work were approved by the Italian Ministry of Health (authorization n. 78/2017-PR) in accordance with the guidelines on the ethical use of animals from the European Community Council Directive of September 22, 2010 (2010/63/EU), and from the Italian D.Leg 26/2014.

Note that full information on the approval of the study protocol must also be provided in the manuscript.

Human research participants

Policy information about studies involving human research participants

Population characteristics	The characteristics of all patients were described in table 1 and 2.
Recruitment	Patients were recruited from the Rare Neuromuscular Diseases Centre of Umberto I Hospital in Rome. Informed consent was obtained from all the subjects. Patients were evaluated using the ALS Functional Rating Scale-Revised (ALSFRS-R), the Medical Research Council (MRC) score. Post-mortem material was obtained at autopsy from 12 ALS patients at the department of (Neuro)Pathology of the Amsterdam UMC, Academic Medical Center, (University of Amsterdam, the Netherlands). All patients fulfilled the diagnostic criteria for ALS (El Escorial criteria45) as reviewed independently by two neuropathologists. All patients with ALS died from respiratory failure. Control spinal cord tissue was obtained from 8 patients who had died from a non-neurological disease. Both ALS and control patients included in the study displayed no signs of infection before death. Since all the patients were selected for ALS diagnostic criteria fulfilment, we believe that there are no selection biases in our results.
Ethics oversight	For patients in Rome, from the Ethic committee of Umberto I Hospital in Rome. For patients in the Netherlands, informed consent was obtained for the use of brain tissue and for access to medical records for research purposes and approval was obtained from the relevant local ethical committees for medical research.

Note that full information on the approval of the study protocol must also be provided in the manuscript.

Flow Cytometry

Plots

Confirm that:

- [x] The axis labels state the marker and fluorochrome used (e.g. CD4-FITC).
- [x] The axis scales are clearly visible. Include numbers along axes only for bottom left plot of group (a 'group' is an analysis of identical markers).
- [x] All plots are contour plots with outliers or pseudocolor plots.
- [x] A numerical value for number of cells or percentage (with statistics) is provided.

Methodology

Sample preparation	Immune cells from wt or hSODG93A mice were enriched by centrifugation on percoll 40%, washed in PBS and immunostained with fluorochrome-conjugated anti-CD3, anti-NK1.1, anti-CD19 and anti-CD45.2 to identify T cells (CD3+NK1.1-) NK cells (NK1.1 +CD3-) and B cells (CD19+). To determine intracellular IFNγ production, cells were maintained in colture for 6 h in the presence of Brefeldine A (10µg/ml), stained with anti-CD56 and -CD3 and subsequently fixed and permeabilized using Cytofix/cytoperm kit (BD Biosciences). After permeabilization, cells were stained with anti-IFN-γ specific mAb and analyzed by FACS.
Instrument	FACSCanto II (BD Biosciences).
Software	FlowJo Version 9.3.2 software (TreeStar).
Cell population abundance	Purity of the sorted cell fractions was confirmed by flow cytometry resulting in a purity of the sorted cells of >98%., as determined by reanalysing by FACS a fraction of sorted cells.
Gating strategy	One supplementary figure (S7) provides information for FACS gating strategies.

- [x] Tick this box to confirm that a figure exemplifying the gating strategy is provided in the Supplementary Information.
